# Ready and Healthy for Kindergarten: A Collaborative Multilingual Family Involvement Program Created by Teachers, Pediatricians, and Parents

**DOI:** 10.3390/educsci15020209

**Published:** 2025-02-10

**Authors:** Lucia Lakata, Lesley Mandel Morrow, Maria Lattmer, Silvia Perez-Cortes, Usha Ramachandran, Shilpa Pai, Daniel Lima, Alicja Bator, Benjamin F. Crabtree, Maria B. Pellerano, Pamela Ohman Strickland, Nila Uthirasamy, Kevin Guerrero, Caroline Mendoza, Manuel E. Jimenez

**Affiliations:** 1 The Department of Pediatrics, Rutgers Robert Wood Johnson Medical School, New Brunswick, NJ 08901, USA;; 2 Graduate School of Education, Rutgers University-New Brunswick, New Brunswick, NJ 08961, USA;; 3 The Department of World Languages and Cultures, Rutgers University-Camden, Camden, NJ 08961, USA;; 4 The Department of Family Medicine and Community Health, Rutgers Robert Wood Johnson Medical School, New Brunswick, NJ 08961, USA;; 5 Department of Biostatistics and Epidemiology, Rutgers School of Public Health, Piscataway, NJ 08961, USA;; 6 Children’s Specialized Hospital, New Brunswick, NJ 08961, USA

**Keywords:** collaboration, partnerships, kindergarten readiness, early literacy, health routines, family involvement, multilingual Spanish education, child health

## Abstract

Ready and Healthy for Kindergarten is a Spanish/English multilingual family involvement program that is grounded in sociocultural and family literacy theories since it focuses on health and literacy development. The program’s development reflects a collaborative partnership of teachers, pediatricians, families, and an advisory board of multilingual parents dedicated to preparing preschoolers and their families for kindergarten. Health themes are presented to introduce foundational literacy and social-emotional skills, and the program comprises eight one-hour virtual sessions intended to engage both children and their families through active participation in learning. The program highlights how parents, educators, and pediatricians can come together to align their expertise and impact family knowledge around child development needs. With this strong collaboration when designing and implementing the Ready and Healthy for Kindergarten program, we were able to successfully provide guidance and support for families, which helped them to establish routines that promote health and well-being, along with some literacy skills.

## 1. Introduction

This article shares lessons learned from implementing Ready and Healthy for Kindergarten as a partnership-centered program supporting the literacy and health routines of Latino families.

A father looks puzzled as an online class begins. This is his first time participating in the class. His preschool-aged son, aware that his father has never attended before, reassures him not to worry because he will explain what is happening. Based on the sessions he attended with his mother, the son explains how the class activities will unfold. He leads his father through the session, and they share these moments joyfully.

Every week, for eight weeks, Ready and Healthy for Kindergarten participants, like the child and father in the vignette, log in to a Zoom session with their teacher, ready to discuss a new health topic, listen to a related story, and learn the corresponding letters/sounds and words together. Each session follows a predictable structure to ensure that the children and their families are confident and engaged in learning. The program was developed as a partnership between teachers, pediatricians, and families grounded in socio-cultural and family literacy theories.

The field of literacy needs to embrace the concept that “it takes a village” (Clinton, 2006) for literacy instruction to be successful. When teaching reading, we must include teachers, parents, and community resources (Pearson et al., 2023; Duke & Purcell-Gates, 2003). Literacy learning takes place in various settings, embodying multiple repertoires of knowledge, dispositions, and ways of using language that are embedded in routine cultural practices. Therefore, the complex nature of learning to read must involve more than the school setting. Families, educators, health professionals, and other community resources can and should play a role in literacy development, beginning at birth. This idea reflects sociocultural theory: every child is unique and comes to school with a different schema based on their cultural and socio-economic background (Moll, 1994; Rosenblatt, 1978). When we speak of family literacy theory, we are referring to the ways in which families and their children use literacy at home and in their community on a regular basis (Taylor, 1983). A family involvement program is one in which children and families participate, children’s socioeconomic and cultural backgrounds are acknowledged, and children and their families engage in experiences that will enhance literacy development. When developing such programs, we need to be aware that although different literacy experiences may not align with the goals of a school’s Language Arts program, we must view cultural and linguistic differences as valuable opportunities to be embraced rather than problems to be solved. This requires the development of a complex and multifaceted relationship between culture and learning, and knowledge about the backgrounds of the children in our classrooms. In other words, we need to practice culturally responsive teaching (CRT) to make literacy relevant for the families we serve.

CRT is grounded in a belief that differences among ethnic and cultural groups are normative to the human condition and valuable to personal and societal development. According to Ladson-Billings (2021), CRT includes (1) literacy for academic success (teaching skills, knowledge, and proficiencies), (2) literacy as cultural competence (teaching in ways that honor and affirm students’ cultures), and (3) literacy as sociopolitical consciousness (teaching in ways that connect to the real world, helping students to apply academics to solving problems related to marginalization and inequities). Parental involvement literacy programs, when defined in this way, employ multiple perspectives for learning related to cognition, the sociocultural environment, and theories related to power and justice.

Family involvement programs (different from family literacy, which is the literacy that already exists in the home) seek to enhance literacy and help develop rich literacy environments for children. Research related to the value of family literacy programs has been carried out at many grade levels and with different populations of students. Large-scale investigations by Henderson and Mapp (2002) and Houtenville and Conway (2008) found that parental involvement in their children’s literacy development from early childhood resulted in students with higher grades, better social skills, lower rates of retention, and higher rates of high school graduation and post-secondary study.

Intentional outreach engages and empowers families early in children’s academic journeys. Cross-disciplinary partnerships offer an important opportunity to build relationships with families (Morrow, 1995). Together, they also represent ideas from different cultures. Both pediatric healthcare professionals and educators identify school readiness as a priority. In fact, the American Academy of Pediatrics prioritizes school readiness as a focus for 4- and 5-year-old well-child visits because it is a critical upstream determinant of health (Hagan et al., 2017). Although parents are a child’s first teachers, primary care involves near-universal access to young children before the age of 5, frequent contact with their families, and opportunities to build trusted relationships (Williams et al., 2019). For example, Reach Out and Read is a model pediatric intervention that seeks to ensure that every family receives a book and guidance about literacy development, along with clinician modeling of shared reading during well-child visits (Needlman et al., 2005). These opportunities make pediatric professionals an ideal partner for educators. However, cross-disciplinary education–healthcare partnerships are relatively uncommon.

We designed the Ready and Healthy for Kindergarten program to build partnerships between educators, pediatric professionals, and an advisory board of families, and to draw on their unique expertise to support children holistically. Ready and Healthy for Kindergarten (RHFK), a Spanish/English multilanguage family health and literacy program, prepares preschoolers and their families for their kindergarten year and beyond by using health topics to introduce foundational literacy and social-emotional skills. The program consists of eight one-hour sessions that can be virtual or in-person and engage both children and families through active participation. Following a predictable session routine to maximize the ease of participation, RHFK is designed to support both heritage language maintenance and the acquisition of the majority language. Families engage in reading aloud, songs, movement, and discussions to learn or review targeted letters, sounds, and vocabulary words using the health themes of Exercise, Nutrition, Bedtime Routines, and Social/Emotional Development. As such, the program’s content reflects parent, educator, and pediatric involvement.

## 2. Instructional Context

### 2.1. Participants’ Background

RHFK participants included Latino families with children between the ages of four and six who were entering kindergarten. The program can take place in the summer or as a beginning unit in kindergarten.

### 2.2. Instructional Setting

We initially designed RHFK as an in-person program (Shelton et al., 2021) but transitioned to an online format during the COVID-19 pandemic (Choi et al., 2023). We found that the online program could involve families living at considerable distances from each other and include the parents at times that did not interfere with work or other family routines. Currently, sessions are held online once per week for eight weeks. Each session lasts approximately one hour, with children and their caregivers participating together. Facilitators of the program are multilingual, certified early childhood teachers who lead the children and adults through a predictable instructional routine using visual supporting material and resources that are provided for the families. Facilitators use the families’ native language to establish connections with them and to foster inclusivity and engagement. Teachers receive a manual and undergo professional development before delivering the sessions.

Text messages and emails containing the links to join the sessions are sent to parents, who also receive support if they experience technical issues in connecting to the sessions. They also receive guidance on how to set up an area in their home for optimal learning (e.g., ensuring that the child is seated at a table or other surface and that the room is as distraction-free as possible). Adults are physically present, sit next to their children, and have all their materials ready.

## 3. Program Components

The program follows a thematic approach to teaching and learning for young children. Thematic instruction integrates content and develops skills in a meaningful way that makes sense to children (Morrow, 2020). The themes focus on health issues, specifically, Exercise, Nutrition, Bedtime Routines, and Social/Emotional Development. The themes engage children and their families in learning and encourage them to discover literacy-promoting activities in everyday health routines.

### 3.1. Theme Content

The sessions encourage active participation and contributions from the children and their families, who are encouraged to share their existing routines related to each theme, as the program’s intention is to empower families by building upon their existing strengths. Families discover literacy and language-promoting activities already embedded in their existing routines (e.g., meal preparation) while being offered suggestions on how to further promote academic readiness and family wellbeing concurrently (e.g., shared reading).

### 3.2. Exercise Theme

Families discuss various forms of physical activity and set daily goals to become more active as a family. They learn how physical activity improves attention and alertness and fosters new cell growth and connections in important areas of the brain (Ratey, 2008).

### 3.3. Nutrition Theme

Families discuss the value of sharing meals (even snacks, if work schedules conflict with traditional meals) and the importance of fruits and vegetables. They set goals for sitting down to eat together and aim for two to three servings of fruits and vegetables daily. Families are encouraged to include children in meal preparation (e.g., choosing ingredients for family meals) and are given information on where to purchase affordable produce.

### 3.4. Bedtime Routine Theme

Families discuss the importance of predictable bedtime activities like brushing teeth and reading a book before going to sleep. The sessions review the important role that sleep plays in cognitive development and offer guidance on how many hours of sleep children need. Dental hygiene and routine visits to the dentist are also highlighted.

### 3.5. Social/Emotional Theme

Families discuss strategies for teaching children how to manage “big” feelings. Both children and adults discuss self-regulation and are introduced to breathing exercises they can perform together. This theme also focuses on the role that identity plays in social/emotional well-being and involves families in discussing the importance of their heritage language and cultural customs and the advantages of being multilingual. While these health themes serve as the content for the sessions and are supported through thematic literature and activities, literacy development is embedded in the themes. This provides a reason for learning the skills that are presented.

### 3.6. Literacy Skills

The program provides experiences with phonemic awareness, phonics, learning the alphabet, vocabulary development, and handwriting. The literacy in the program is not meant to be a systematic, direct instruction of these skills. The tasks are meant to introduce parents and children to literacy experiences and activities. The skills are first taught and then embedded into the stories, songs, and themes in the program (Shanahan, 2025; Blevins, 2024). This is not the direct and systematic instruction of word study skills that will happen in kindergarten on a regular basis for about 20 min daily.

Reading and listening comprehension are addressed, through interactive reading aloud with discussions and teaching about story retelling. All books used in the program support the four health themes and provide families with examples of high-quality, age-appropriate literature. Selected picture books reflect the work of multicultural authors, popular children’s book series, and classics with rich language and good story structure in addition to alignment with the health themes. When selecting books, we considered the availability of books in both English and Spanish to support bilingual literacy development and to maximize parental involvement. Most parents who participated in the program had English as a second language and felt more comfortable reading in Spanish.

During interactive reading aloud, the teacher models excellent storybook-reading practices by mentioning concepts about books and print, asking and answering questions, and reinforcing story retelling. Families actively participate by retelling the story with their children using specific transition words and prompts (e.g., “first”, “next”, “then”, “after that”, and “finally”). Encouraging families and children to retell stories offers active participation that helps develop language structures, comprehension, and a sense of story structure (Morrow, 2005). Families are encouraged to retell in either Spanish or English. This is also a time to discuss the theme of the book and what can be learned from it. The books for each theme, along with their featured word and letter, appear in Table A1.

Children were introduced to the selected words from the themes. We selected words that could be pictured and acted out; for example, the child pretends to be brushing their teeth for the word **Toothbrush** in the unit on **Bedtime Routines**. The word **Toothbrush** appears in the book read for that theme; in this case, it is *How do Dinosaurs Go to Sleep?* Jane Yolen (2016, Blue Sky Press) (in English).

To teach the letters and sounds, the children are led through routines, including the teacher saying the letter, sounding the letter, and reading picture cards for the word. In addition, children practice writing the target letter. The letters, sounds, and words are taught to children in both English and Spanish. For example, in the theme of bedtime routines, one of the books is *Dulces Sueños/Sweet Dreams* by Pat Mora. The word **Bed** is the focus of this session, as the story features a grandmother and her grandchildren discussing the different things that they do to get ready for bed. In this session, the children learn the sound of **B**, write the letter **B** on a whiteboard, read the word BED on a picture card, and find it in the book. The target word and its first letter are presented in English and Spanish (e.g., **cama**). An important part of the literacy development instruction provided in the sessions is to bring awareness of the similarities (and contrasts) between English and Spanish, particularly at the phonemic level. For example, while the sound of **B** is quite similar in both languages, the letter **H** in the word **Hug**, introduced in the theme about social and emotional health, is not pronounced in the same way in English and Spanish. The program includes a review of sounds that present both similarities (e.g., /b/, /c/) and differences (e.g., /r/, /h/) in how they are articulated in English and Spanish. The selection of words was dictated by the content of the books. The goal of highlighting such cross-linguistic comparisons was to encourage parents, as well as children, to see their multilingualism as a resource and an asset. Reinforcing the importance of continuing to build an understanding of the heritage language as children continue to be exposed to English is a theme throughout the program.

RHFK engages children in handwriting, using the first letter in a word that is the focus of a particular theme. Handwriting directly supports the writing-to-reading process, and practice is key to enhancing fine motor control in order to form letters. Teachers guide writing, for example, when learning to write the letter “**h**” for the word **Hug** in the Socio-emotional theme, children are encouraged to say, “You start at the top and go down, and then write one hump to form the **h**”.

### 3.7. Predictable Structures to Promote Social-Emotional Readiness

Children, and adults, thrive on predictable routines, which promote independence, responsibility, and a sense of order. The sessions follow a predictable routine: (1) welcome, (2) singing the alphabet song, (3) interactive reading aloud/vocabulary, (4) a theme-related song, (5) letters/sounds, (6) an exercise/movement break, (7) health content, (8) an at-home activity review, and (9) wrap-up. The program manual prompts the facilitator to explicitly unpack these structures for families and explain their significance (Table A2). For example, before movement breaks, the facilitator states that such breaks can help children refocus during long or complicated tasks. The predictable routines in this program promote self-guidance and self-regulation (Manfra & Winsler, 2006). This concept can become a tool for parents to utilize with their children at home.

## 4. Implications for Practice

Community partnerships can enhance outreach and facilitate access to high-quality support for young Latino multilanguage learners and their families. Specifically, RHFK highlights how parents, educators, and pediatricians can align their expertise to simultaneously promote academic and physical readiness for school. RHFK engages families through interactive discussions. It recognizes the importance of home language and builds on these strengths. Through its parent advisory board, parents play a leadership role in setting the direction of the program.

RHFK leverages health routines that pediatric clinicians have identified as important for physical readiness for school (i.e., sleep, nutrition, exercise, and social-emotional development) and as a platform for families to discover the literacy-promoting activities naturally embedded in those routines (e.g., reading nutrition labels). RHFK uses these themes as a vehicle to encourage families to practice letters/sounds with their children, read together, and practice retelling stories that will enhance academic readiness. We have successfully established these partnerships and found that all partners make important contributions to the work.

This type of partnership might seem difficult to initiate. However, it can be easily accomplished. There are thousands of **Reach Out and Read** programs throughout the country (often associated with university hospitals). In addition, over 27 million families nationwide are served by community health centers (CHC), which can initiate and host family literacy program partnerships to promote school readiness in underserved communities.

Zoom gives us the ability to form partnerships with parents and children more easily than ever before. Meetings between pediatricians, parents, and teachers are easy to hold. Schools could use RHFK as a family involvement program at the end of the preschool year or in the summer before kindergarten. This can also be a family involvement program during the kindergarten year, held in the early evening once a month from September through May, either live or on Zoom. Teachers and pediatricians can initiate this type of collaboration, and the program can also be delivered solely in English.

Schools and pediatricians need to partner with programs such as the one described here. At this time, we are sharing our RHFK program with other communities, and we hope that it will be replicated so that all children, especially those in marginalized communities, have access to programs for health and academic readiness for school, thereby promoting equity in academic achievement and wellbeing.

## Data Availability

The original contributions presented in this study are included in the article. Further inquiries can be directed to the corresponding author.

## Appendix A

**Table A1.** The themes, books, letters, and vocabulary introduced in each unit.

There are two books per theme. In the first week, the storybook is in Spanish and is read in Spanish. The next week, the storybook is in English and is read in English.
**Workshop 1: Exercise**
**Book:** *De la cabeza a los pies* (From Head to Toe), Eric Carle (2003), Harper Collins (in Spanish) **Letters introduced:** w **Vocabulary words introduced:** wave (saludar)
**Workshop 2: Exercise**
**Book:** *Are You Ready to Play Outside?* Mo Willems (2008), Hyperion (in English) **Letters introduced:** r **Vocabulary words introduced:** run (correr)
**Workshop 3: Nutrition**
**Book:** *La oruga muy hambrienta,* Eric Carle (1994), Random House (in Spanish) **Letters introduced:** s **Words introduced:** strawberry (fresa)
**Workshop 4: Nutrition**
**Book:** *Growing Vegetable Soup,* Lois Ehlert (1987), HMH Publishers (in English) **Letters introduced:** c **Words introduced:** carrot (zanahoria)
**Workshop 5: Bedtime Routines**
**Book:** *Dulces Sueños/Sweet Dreams,* Pat Mora (2008), Harper Collins (in Spanish) **Letters introduced:** b **Words introduced:** bed (cama)
**Workshop 6: Bedtime Routines**
**Book:** *How Do Dinosaurs Go to Sleep?* Jane Yolen (2016), Blue Sky Press (in English) **Letters introduced:** t **Words introduced:** toothbrush (cepillo de dientes)
**Workshop 7: Socio-emotional Health/Behavior**
**Book:** *Un Beso en Mi Mano* (The Kissing Hand), Audrey Penn (1993), Tanglewood Publishing (in Spanish) **Letters introduced:** h **Words introduced:** hug (abrazo)
**Workshop 8: Socio-emotional Health/Behavior**
**Book:** *Where Are You From?* Yamile Saied Méndez (2019), Harper Collins Publishing (in English) **Letters introduced:** l **Words introduced:** love (amor)

**Table A2.** Examples of notes to parents from the teaching manual.

**Note to parents:** Point out the *turn and talk* picture on the slides. Let parents know that every time they see this image moving forward, it means they will engage in a turn and talk with their child.
**Note to parents:** Highlight the fact that breaking up activities/tasks/homework with physical activity or eating a healthy snack is a good habit to help children remain engaged. Physical activity during break time counts toward the 60-min daily goal. Also, remind families that dancing is a form of exercise that can be a great break for children.
**Note to parents:** Let families know how important it is to point out language similarities and differences to children, and also, how important it is for them to continue supporting the children in their home language so that they can transfer their learning to the new language.

**Table A3.** Descriptions of at-home activities.

Activity Book Tasks	At-Home Extension Activities (One per Week)
Handwriting practice Drawing objects that begin with the focus letter Thematic song lyrics Reminder to reread the book of the week and practice retelling Discussion prompts with sentence stems to support conversations about the health theme Letter/sound/picture cards	Create a choice board with different types of exercise and choose one a day to do as a family (exercise) Make a list of different games to play inside and outside (exercise) Find pictures of healthy foods and treats and sort them by category (nutrition) Pick a green fruit or vegetable to add to one meal, then draw/write about it (nutrition) Make a list using words and pictures of bedtime routine (bedtime routines) Pick a favorite book to read and read that same book every night before bed for a week (bedtime routines) Draw/write about something to do when worried or upset (social and emotional) Draw a picture of someone in the family who is brave (social and emotional)

**Table A4.** Sample pages from at-home resources.
